# Effects of LED lighting on the nutritional properties and microbial safety of microgreens

**DOI:** 10.3389/fnut.2026.1869208

**Published:** 2026-06-24

**Authors:** Anushuya Guragain, Zeynal Topalcengiz, Juan Moreira

**Affiliations:** 1 Department of Food Science and Human Nutrition, Colorado State University, Fort Collins, CO, United States; 2 Department of Food Engineering, Faculty of Engineering and Architecture, Muş Alparslan University, Muş, Türkiye

**Keywords:** LED light, nutritional properties, pathogens, PPFD, wavelength

## Abstract

Microgreens are immature and nutritious seedlings, rich in micronutrients and bioactive compounds these crops are commonly grown in controlled environments. Various light sources are used during indoor growth of microgreens depending on the specific light spectrum requirements of the crop, the energy efficiency of the lighting system, and budget-oriented market availability of production systems. Light Emitting Diode (LED) treatments offer a promising opportunity for microgreen production. This review synthesizes current literature on the effects of various treatments/conditions, emphasizing LED light spectra (wavelength), intensity, and photo period on microgreen characteristics including yield and growth, nutrient composition, and microbial safety. Currently, research on lighting effect on nutritional properties focus on Brassicaceae family which could be further expanded to other plant groups. Also, limited research on photoperiod (LED lighting duration) represents a research gap. Influence of the light quality, intensity and photoperiod have been reported on microgreen growth, yield and nutritional properties which vary with microgreen type. Although foodborne pathogens are known to persist and proliferate in microgreens under poor production and handling conditions, there remains a critical research gap regarding microbial survival, proliferation, and antimicrobial efficacy during production and storage under LED lighting systems. This highlights a need for comprehensive research to develop food safety protocols for microgreen production with LED light treatment holding significant potential.

## 1 Introduction

**FIGURE 1:**
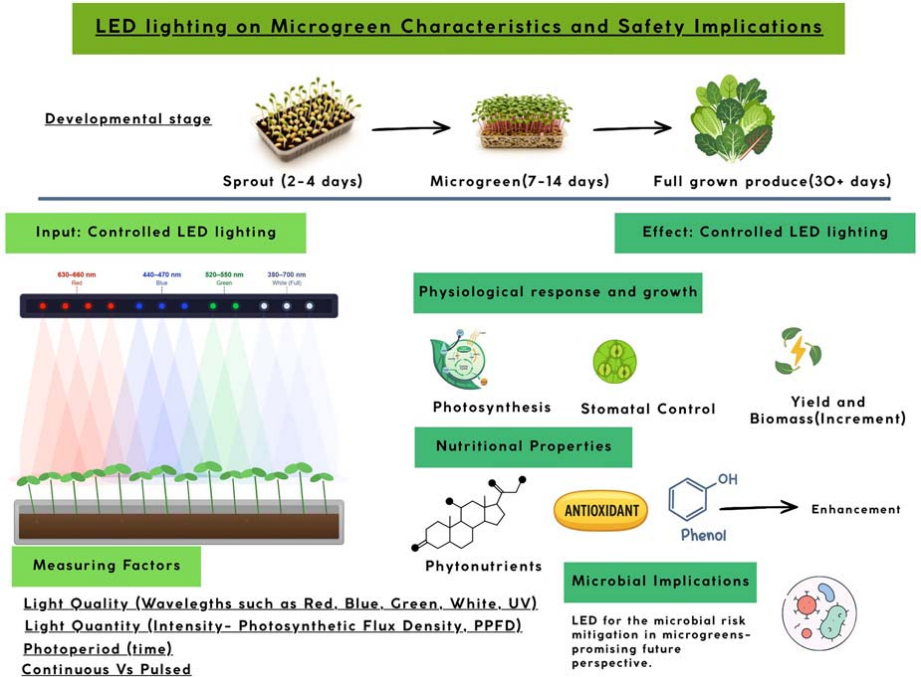
Measurable parameters from LED Lighting, and the different characteristics impacted on microgreens.

The market for fresh and functional foods is expanding, driven by the growing interest of consumers in diets that actively enhance well-being and longevity. Essentially, there is an increasing demand for food that provides benefits beyond basic nutrition (1). As societies shift toward healthier food habits, there has been a global surge in demand for fresh and ready-to-eat functional food such as microgreens and sprouts which have various health promoting factors including rich phytonutrient contents (2, 3). Microgreens are immature seedlings cultivated from a variety of crops (including vegetables, herbs, and grains) and harvested 7–14 or 21 days after germination when true leaves appear within a diverse range of flavors, colors, textures, and aromas (4). Prior to the development of true leaves and after formation of cotyledons these same crops are considered sprouts (Figure 1).

The global microgreens market is projected to rise from USD 2.55 billion in 2025 to USD 6.81 billion by 2033 with a compound annual growth rate (CGAR) of 13.1% during the forecast period 2024–2031 (5). The upward trend is mirrored in North America, where the market is expected to reach USD 1,503 million by 2031, reflecting a compound annual growth rate of 10.1% over a decade (6). The United States is a significant contributor of the global microgreen market, while Canada and Mexico also have substantial shares (7). Within the United States, demand for microgreens is primarily fueled by culinary purposes where they are used to enhance flavor profiles, and garnish various culinary inventories such as salads, soups, plates and sandwiches (8, 9).

Studies have consistently reported that microgreens possess a significantly higher concentration of phytonutrients, approximately 30–40 times higher concentration than mature plants (7, 8, 10, 11). The nutritional profile, including phytonutrients of sprouts and microgreens generally surpasses that of ungerminated seeds or their mature counterparts (3). For instance, the microgreen cotyledon leaves including red cabbage, cilantro, garnet amaranth, and green daikon radish were found to be significantly more nutrient-dense than mature plants listed in United States Department of Agriculture (USDA) National Nutrient Database (10). These prominent differences compared to mature greens highlight the potential of microgreens to serve as an exceptionally concentrated source of dietary phytonutrients.

While there have been no recorded incidents linking microgreens to foodborne outbreaks like that of sprouts, they have recently been subject to several recalls in America and Canada because of *Salmonella* spp. and *Listeria monocytogenes* contamination (12, 13). The microgreen production safety system can be compromised by a variety of transmission routes which could be a potential entry point for foodborne pathogens. Pathogens may be introduced through multiple routes in the production cycle, including but not limited to seed, water quality, growth medium (for instance, soil vs. hydroponic substrate), environmental factors, pre -harvest and post-harvest handling practices during transfer and storage (14–18). Once contaminated, various types of microgreens may favor survival of foodborne pathogens until consumption (19–22). The short shelf life of microgreens, coupled with survival and proliferation of foodborne pathogens during their production cycle has led to concerns from the industry and has driven studies to the exploration of post-harvest treatments (19).

The cultivation of microgreens spans a broad spectrum of complexity, from highly automated controlled environment agriculture to primitive, low tech home environments (23–25). While small-scale controlled environment agriculture units offer low-cost flexibility and rely heavily on manual oversight, industrial-scale infrastructure utilizes full automation to maximize yield uniformity across thousands of trays. Over time, protected cultivation has evolved from basic, light-permeable greenhouse setups into sophisticated high tech plant factories commonly known as Controlled Environment Agriculture (CEA) or Controlled Environment Plant Production System (CEPPS) (26). CEA is becoming the standard for microgreen production with the focuses on efficient use of resources such as energy, water and fertilizers (27). Modulating or regulating the quality/wavelength, quantity/intensity over time and duration of light allows producers to significantly influence the color, flavor, nutritional content, aroma and quality of microgreens (2).

Use of artificial lighting systems such as Light Emitting Diode (LED) have been growing in recent years in fresh produce, sprouts and microgreen production (4, 28). These systems typically utilize LED lighting providing precise control over light spectrum and intensity with reduced heat radiant emissions (29). Optimizing microgreen production involves selecting from a diverse light spectrum–including red, blue, green, white, yellow, and red-blue combinations. Additionally, ultraviolet LEDs (UV-A, UV-B, and UV-C) are emerging as supplementary lighting to stimulate secondary metabolism, trigger photoprotective responses, and enhance phytonutrient accumulation (30–32). Various studies have examined the impact of intensity of LED light (such as red, blue and white) on microgreen yield, growth and nutritional properties including phytonutrients such as phenolics, carotenoids, and glucosinolates (13, 30, 33). For instance, red, blue and their combined spectra are in general more effective than white light or other wavelength at driving photosynthesis and regulating plant metabolism (30), whereas the impact of same light quality on a plant’s nutrition and biochemistry can change significantly depending on its intensity (13). Therefore, maximizing both the yield and functional quality of microgreens requires a precise calibration of both wavelength selection and the specific irradiance levels tailored to the target cultivar.

While individual studies exist, a comprehensive review focusing on the dual effects of LED illumination on both microgreen food safety and nutritional quality is currently lacking in the literature. This review synthesizes current knowledge on artificial lighting systems with a focus on LED systems in microgreens to analyze nutritional properties such as phytonutrients, quantitative properties such as yield and growth, and food safety implications. This review outlines available cultivation infrastructure and details how light quality, intensity, and photoperiod modulate crop characteristics. Finally, the manuscript synthesizes how LED technology as a light source during microgreen production affects nutritional biofortification and microbial safety intervention to control foodborne pathogens.

## 2 Lighting systems in microgreen production and storage

Microgreen producers often supplement or entirely replace natural sunlight with artificial lighting to ensure consistent yields, to avoid plant diseases, to enhance phytonutrients and to grow relatively safe crops in controlled environments. Since solar radiation in greenhouses is frequently unreliable due to weather shifts and seasonal changes, artificial lighting systems are essential for maintaining stable growth environments, yield and enhancing the plants’ nutritional profiles (4, 34). CEA technologies with artificial lighting systems such as greenhouse, aeroponics, hydroponics, aquacultures as well as the vertical farming possibility offers promising alternative and complementary sources to traditional farming. These technologies enable reliable food production in regions with harsh climates, limited sunlight, or scarce land, ranging from disaster prone areas and saline soils to urban centers and space stations (35, 36).

Initial studies on artificial lighting systems in microgreen production show use of traditional cold and white, and incandescent fluorescent lamps (high-pressure sodium light bulbs–HPS). Given the spectral range of HPS lamps, research suggested that HPS alone may not provide the full range of wavelengths necessary to meet the physiological and developmental requirements of plants which prompted researchers to initiate studies aimed at enhancing the lighting environment by supplementing HPS lamps with LED sources, which offer greater flexibility in spectral tuning and can potentially address the deficiencies of HPS lighting (37, 38). Research on substituting HPS lamps with LED lightings was initiated after it was noted that HPS produced significant energy as thermal radiation and raised temperatures on the lamp surface (38, 39). As a result, in recent years, there has been a notable surge in both scientific research and technological development focused on LED lighting systems.

## 3 The role of light and lighting systems during growth of microgreens

In photosynthesis, red light is essential for the functioning of the photosynthetic apparatus and the accumulation of biomass by driving CO_2_ fixation and reducing intercellular CO_2_ concentrations (40, 41). Conversely, blue light is qualitatively required for chloroplast development and phytochemical formation, but it also serves as a regulatory switch that can induce stomatal closure via the dephosphorylation of H^+^- ATPase pumps (42). Considering, high fluence red light can lead to poor morphological development as a result of fast photodestruction of a newly formed Chlorophyll (Chl) molecules in plants (35), it is recommended to integrate additional lighting spectra throughout the growth process (34). The photosynthetic processes of plants grown under controlled artificial lighting can be strategically modified by adjusting the quality of light they receive. For instance, plants cultivated under monochromatic blue or red LED light, often exhibit significantly higher synthesis and accumulation of secondary metabolites than when grown under traditional white light sources, suggesting that specific light spectra can enhance specific biochemical pathways (43–45).

Several studies have focused on the influence of specific LED light spectra (wavelength or quality) on the photosynthetic efficiency of microgreens. Various LED treatments in microgreens with key findings are shown in Table 1. For example, adding green LEDs to blue + red LEDs can enhance plant growth, but only in moderation. Keeping green light under 24% of the spectrum has been shown to enhance development in some species, whereas exceeding 50% reduces plant growth (46). While red and blue light are mostly absorbed in the upper part of the canopy, supplementing green and far-red light may be beneficial as the leaf area increases which could be better utilized in the lower leaves (47, 48). A multi-spectral approach that carefully regulates the proportions of red, blue, and supplemental light is essential to sustain healthy microgreen physiology and canopy development.

**TABLE 1:** Effect of LED and different lighting systems on microgreens characteristics.

Microgreens	Lighting type/system	Growing conditions	Key findings (effect on microgreen characteristics including nutrition, yield, growth)	Reference
Red pak choi (*Brassica rapa* var. chinensis), mustard (*Brassica juncea* L.), tatsoi (*Brassica rapa* var. rosularis)	Pulsed-LED lighting consisting of HPS lamps supplemented with monochromatic LEDs	Grown in an environmental growth chamber, day/night temperatures of 21/17 °C, and 16/8 light/dark photoperiod. Relative humidity of 5%.	Enhanced anthocyanin accumulation. Impacted antiradical scavenging activity (increase or decrease according to wavelength)	(59)
Kohlrabi (*Brassica oleracea* var. gongylodes), broccoli (*Brassica oleracea*) and mizuna (*Brassica rapa* var. Japonica)	Combinations of LED light spectra (blue light 447 nm, red at 638 nm and 665 nm, far-red at 731 nm), supplemented with either green light (520 nm), yellow (595 nm) or orange (622 nm)	Grown environmental chambers using peat substrate in 0.5 L plastic pots for 10 days. Day/night temperatures 21/17 °C. Photoperiod was 16 h, with relative humidity of 50%–60%.	Induced metabolic changes that in turn lead to increases in essential nutrients, including Fe, Mg, and Ca.	(60)
Mustard (*Brassica juncea* L. “Florida Broadleaf”)	After emergence of true leaves, microgreens were treated with either 275 μmol photons m^–2^ s^–1^ or 463 μmol photons m^–2^ s^–1^ until a total of 36 h of photoperiod	Polyethylene teraphtalate pads were used to germinate and grow microgreens. Pads were placed in perforated trays, which were then set in solid-bottom trays. Experiments performed in environmental chambers, with an initial photoperiod of 14 h under 275 μmol photons m^–2^ s^–1^	Increased concentration of carotenoids, zeaxanthin, and antheraxanthin. Reductions in chlorophyll b.	(70)
Kale (*Brassica oleracea* L. var. acephala)	LED light irradiation with white light (450–660 nm) blue light (450 nm) or red (660 nm) with a flux rate of 90 μmol m^–2^ s^–1^	Grown in an environmental growth chamber at 25 °C, in plots containing vermiculite. Photoperiod light/dark 16/8 h.	White light irradiation led to higher carotenoid content, blue light increased phenolic content and antimicrobial activity. White and blue both increased glucosinolate in microgreens.	(57)
Radish (*Raphanus sativus* L)	LED irradiation using either white or red:blue light at a 15 cm distance. UV-C irradiation (254 nm), at 40 cm distance from the lamp, and during one of four different times (5, 10, 15, or 20 min)	Microgreen seeds incubated in trays for 3 days, at 27 °C, under dark conditions. After germination microgreens were maintained at room temperature.	Microgreens grown under LED and later exposed to 10 min of UV-C increased chlorophyll, and carotenoid content. UV-C treatment of 20 min was detrimental to quality and nutritional properties.	(32)
Kale (*Brassica oleracea* L. cv “Red Russian), and Mustard (*Brassica juncea* cv. “Red Lace”)	Four-channel LED lighting unit, with a composition of 61% deep red, 20% blue, 15% white, and 4% far red. Three lighting intensities; 150, 200 and 250 μmol m^–2^ s^–1^	Walk-in environmental chamber at 21/17 °C, and 65% relative humidity. Grown 10 days after germination.	Increased α-tocopherol in kale, and β-carotenoid content in mustard microgreens.	(58)
Amaranth (*Amaranthus tricolor*) and Mustard (*Brassica juncea*)	Wide spectrum 4000 K LED light applied (100, 150, 200 and 250 μmol m^–2^ s^–1^). Three light treatments of red, blue and white light were applied.	Temperature of 17/20 °C, and 16 h photoperiod. Microgreens harvested 10 days after germination.	Wide spectrum 250 μmol m^–2^ s^–1^ PPFD along with combination of red and blue light, led to increased sugar content in the microgreens.	(63)
Amaranth (*Amaranthus cruentus*)	Dyna-RHX30 LED lights; using blue (450 nm), green (520 nm), red (660 nm), and broad spectrum white 5700 K lights. Treatments applied at 300 μmol m^–2^ s^–1^ PPFD.	Grown inside a polycarbonate-covered greenhouse. Microgreens sown into mycorrhiza medium with sphagnum peat moss, perlite, vermiculite, and limestone. Maintained under dark for 48 h until germination and prior to light treatment application	Increased vitamin E, vitamin C, carotenoid, sugars, and acid content. Reduction in phenolic compounds and vitamin B.	(67)
Broccoli (*Brassica oleracea* var italica cv. “Ramoso Snatana”)	LED panels (K5 Series XL750) with white (440–660 nm), and far red (730 nm). Six lighting regimes applied with light intensities varying from 50 to 150 μmol m^–2^ s^–1^ PPFD.	Environmental growth chamber, using hydroponic growing mats. Seeds germinated in darkness at 25 °C, and 90% relative humidity for 4 days. Light treatment applied on day 5.	Varying light treatments led to increases in chlorophyll, carotenoid, anthocyanin, ascorbic acid, total phenolics, without far red light included in treatment. Far red also led to increases, but also had decreases in phytochemicals.	(64)
Red cabbage (*Brassica oleracea* var capitata)	LED fixtures (PHYTOFY RL) were placed 43 cm above the samples and extended 41-cm apart for irradiation	Seeds kept under dark conditions at 25 °C, and 90% relative humidity until germination. 16 h photoperiod per day, light treatments applied at 25 °C, and 50% relative humidity.	Blue LED treatments led to larger leaf area, while white LED light led to improved growth. Increases in glucosinolate and polyphenols were also observed.	(65)
Red cabbage (*Brassica oleracea* var capitata)	White LED lamps (Model GLFS-v.11.7.4.22) consisting of blue (25.4%), green (23.8%), yellow/orange (21.3%), red (27.1%), and far red (2.3%).	Carried out in a laboratory, with temperature of 20 °C and a relative humidity of 50%–65%.	315 μmol m^–2^ s^–1^ PPFD led to highest yields and quality of red cabbage microgreens.	(71)

High-pressure sodium (HPS) is a type of gas-discharge light which is increasingly being replaced by other efficient sources like LEDs. Photosynthetic Photon Flux Density [PPFD, μmol/(m^2^ s)] is the amount of light that reaches the plant. Ultraviolet-C (UV-C) is a high-energy, short-wavelength light that is increasingly used as supplemental light source during microgreen production.

Selecting the appropriate type and composition of lighting presents a significant challenge for the optimization of microgreen growth (49). Light conditions, including quality, intensity, and photoperiod, represent critical environmental factors that regulate plant morphology and physiology throughout the entire life cycle (49–51). Light quality refers to spectral composition of the light which is the specific wavelengths (colors) present and/or it’s proportion such as red and/or blue, whereas light intensity represents amount of light reaching the microgreens per time, typically quantified as Photosynthetic Photon Flux Density (PPFD). Moreover, photoperiod is the duration of light exposure within a 24-h cycle and frequency indicate pulsed and continuous light (Figure 1).

## 4 LED lighting for growth optimization and nutritional enhancement in microgreens

Optimization of CEA systems for microgreen production requires a sophisticated understanding of how artificial light functions as both an energy source for photosynthesis and a signaling mechanism for secondary metabolism. Among the variable parameters of artificial illumination, light quality, light intensity, and photoperiod apply the most profound influence on crop development. While spectral quality acts as a qualitative switch that dictates morphogenesis, pigment synthesis, and enzymatic pathways, light intensity functions as a quantitative driver that modulates biomass accumulation, gas exchange, and photooxidative stress responses. Light quality, light intensity, and photoperiod interact closely, meaning their effects on yield, appearance, and nutrient levels depend heavily on the specific microgreen species.

### 4.1 Light quality (spectral wavelengths)

In a CEA facility, light quality or spectral composition strongly influences the accumulation of secondary metabolites in plants. However, mechanisms are not understood clearly, and knowledge on how LED light specifically modulates enzyme activities in microgreens is limited (30). Light quality influences a wide range of plant characteristics, including growth, structural form, pigmentation, flavor, and nutritional composition (2). Red, blue, and a combination of red and blue light spectra are generally more effective than white light or alternative wavelengths for enhancing photosynthetic rates and regulating metabolic processes in plants (30). For instance, vegetables such as pea (52, 53), tomato (54, 55), and Chinese cabbage (56) tend to develop higher phytochemical levels and antioxidant activity when grown under monochromatic red (R), blue (B), or combined (RB) LEDs compared to white (W) LEDs, fluorescent (FL), or high pressure sodium (HPS).

Blue light promotes growth, cotyledon area, fresh weight, chlorophyll a, and anthocyanin content in both red and green basil microgreens; however, phenolic content and free radical scavenging activity have been enhanced by predominantly red light in the green basil cultivar and predominantly blue light in the red basil cultivar (45). Research comparing the effects of LED light spectra (white, blue, red) on kale microgreens found that the white LED light promoted higher carotenoid content, while blue LED light enhanced phenolic content and antimicrobial activity against pathogens, like multidrug resistant *Pseudomanas aeruginosa*. Likewise, white and blue LEDs both slightly increased glucosinolate levels (57). Another study evaluated how different LED cultivation regimes (white vs. red:blue light) combined with post-harvest UV-C irradiation treatments affect the growth, phytochemical content and storage quality of Radish microgreens. The results showed that red:blue LED-grown microgreens exposed to 10 min of UV-C exhibited twice the chlorophyll content of controls, enhanced carotenoid content highest freshness and lowest weight loss after 12 days of storage. However, long UV-C exposure (20 min) was detrimental, leading to the highest weight loss and poorest storage performance (32). In another study, authors found that varying light intensities during growth (deep red 61%, blue 20%, white 15%, and far red 4%) and light exposure during storage significantly affected the phenolic, carotenoid, and tocopherol contents of kale and mustard microgreens, with light storage (White LED) helping preserve their nutritional quality and shelf life (58).

Supplementing HPS lamps with short-term red LED light modified the antioxidant properties of amaranth, basil, mustard, spinach, broccoli, borage, beet, kale, parsley, and pea microgreens. These supplemental light wavelengths increased the production of various bioactive compounds across different species, likely as a protective response to mild photooxidative stress. While total antioxidant activity generally increased, the antioxidant content of amaranth, broccoli, and pea was largely unaffected, and beet microgreens exhibited a decrease in antioxidant levels (37). The effects of continuous versus pulsed LED lighting on phytochemicals levels such as total phenols, anthocyanins, and antiradical activity in three microgreens including red pak choi, mustard and tatsoi of the Brassicaceae family, revealed that pulsed-LED lighting (optimized by frequency) can enhance phytochemical content in microgreens, which may be a useful strategy in CEA production systems, to increase nutritional quality (59). Another study reported that primary LED treatments combining blue, red, and far-red light (447, 638 and 665 nm, 731 nm) or supplemental green (520 nm), yellow (595 nm), and orange (622 nm) for 16 h significantly influenced nutrient and metabolite levels in Brassicaceae microgreens. Results from this study indicate that targeted LED spectrum can be used to modulate nutritional quality, such as enhancing or preserving specific nutrients (60).

### 4.2 Light intensity

The impact of the same light quality can vary significantly depending on light intensity and duration, affecting plant physiology, biochemical pathways, and nutritional outcome (13). The LED light intensity on *Brassica* microgreens reduced nitrate levels and enhanced antioxidants level under moderate irradiance (330–440 μmol m^–2^ s^–1^, PPFD levels), optimizing growth. Meanwhile lower or higher PPFD negatively affected nutritional quality (61). Optimal nutritional quality was observed at PPFD levels of 90 μmol⋅m^–2^⋅s^–1^ for cabbage microgreens and 70 μmol⋅m^–2^⋅s^–1^ for kale microgreens during 16-h d^–1^ photoperiod, whereas increased light intensity led to shorter hypocotyl length for both microgreens (49). *Brassica* microgreens including kohlrabi, mustard, and mizuna grown under combinations of red-blue, red-far-red-blue, and red-green-blue LED spectra at three photon flux densities to achieve daily light integrals of 6, 12, or 18 mol m^–2^d^–1^, evidenced that the impact of light intensity, and quality were species dependent. For instance, total carotenoids decreased as light intensity applied to mizuna and mustard microgreens increased, regardless of light quality. Meanwhile, higher light intensities resulted in increased total anthocyanin content in Kohlrabi (62).

The highest sugar accumulation and improved storage stability in Amarath and mustard microgreens were achieved using a PPFD of 250 μmol m^–2^ s^–1^ along with a spectrum ratio of R88.9%: B11.1%. Therefore demonstrating that a combination of specific cultivation lighting and white LED storage lighting is essential for maintaining post-harvest quality (63). Broccoli microgreens have been found to maintain high yields and quality under low light intensities (50–75 μmol m^–2^ s^–1^), with supplemental far-red light further enhancing plant height and fresh weight. Interestingly, lower light intensity levels also improved post-harvest quality and shelf-life compared to higher intensities (100 and 150 μmol m^–2^ s^–1^), although some phytochemicals, such as total phenolics, were reduced when far-red was added (64). Overall, strategic modification of light intensity and spectrum offers ways to improve both yield and quality of microgreens. Another study found that specific light spectra and intensities enhanced biomass, leaf expansion, and accumulation of phytochemicals, while other combinations had limited or negative effects in red cabbage. For instance, the application of blue LED light increased leaf area and promoted more uniform coloration, whereas white LED light improved growth and appearance. This study highlights that both growth and nutritional traits can be moderated through targeted LED strategies, although responses are highly spectrum- and intensity-dependent (65).

Light intensity significantly influenced fresh and dry weight, stem elongation, chlorophyll levels, and the accumulation of phenolic compounds and antioxidant capacity, with species-specific responses in Brassicaceae microgreens including cabbage, kale, mizuna, and mustard (66). Moreover, mustard and kale microgreens grown under different light intensities, with varied PPFD levels (150, 200, and 250 μmol m^–2^ s^–1^), evidenced that higher light intensities during cultivation and postharvest storage enhanced the antioxidant properties of these microgreens. Likewise, the total phenolic content and specific nutrient compounds such as β-carotene and α-tocopherol, presented a species dependent response (58). Another study investigated the effects of different LED light spectra [red:green:blue (R:G:B) at the ratio of 70:10:20, R:B at 80:20, white (W), or ambient light (greenhouse solar radiation)]. This study concluded that light quality can modulate secondary metabolites, enhance nutritional and functional quality, but optimizing for one compound may reduce others, requiring balanced spectral design (67).

### 4.3 Photoperiod

While it is well established that light intensity significantly impacts plant growth and nutrition, research regarding the effects of photoperiod on plant growth and nutrition is rare. This knowledge gap in data regarding photoperiod is particularly evident in the study of microgreens (13). A study that examined the effects of 10 h and 16 h photoperiods with UV-A LED wavelengths (366, 390, and 402 nm) on mustard microgreens found that lutein/zeaxanthin and β-carotene concentrations peaked under 366 nm (10 h) and 390 nm (16 h), respectively. Additionally, the 402 nm wavelength paired with a 16 h photoperiod yielded the highest accumulation of all minerals, with the exception of iron (68). This result suggests that tailoring UV-A LED light duration can strategically enhance specific phytochemicals and mineral content, but more studies are needed in this area to strengthen this conclusion. Another study found that extending photoperiods from 24 to 48 h increased antioxidant and bioactive compound levels in chia microgreens (69). These findings suggest that photoperiod can be modulated with respect to specific microgreens, to optimize their characteristics such as yield, growth and nutritional parameters (i.e., polyphenols, and antioxidant content) in controlled environment microgreen production.

Adjusting in light management can effectively increase antioxidant and carotenoid levels in mustard microgreens. In a study, plants were grown under controlled conditions using cool white fluorescent (160 W) and incandescent bulbs (60 W) at 275 μmol m^–2^ s^–1^ with a 14-h photoperiod, followed by 36 h of increased light intensity at 463 μmol m^–2^ s^–1^ after the emergence of true leaves from the microgreens (70). The study found that elevated light intensity significantly increased concentrations of carotenoids and other photoprotective pigments, particularly zeaxanthin and antheraxanthin, while slightly reducing chlorophyll b levels and altering the chlorophyll a to chlorophyll b ratio without significantly affecting lutein and biomass yield (70). The effects of harvest time, photoperiod, and white-LED irradiance on the growth and yield of red cabbage microgreens in a plant-factory system suggested that optimizing light intensity, photoperiod, and harvest timing can maximize productivity in controlled environment microgreen production (71).

## 5 Microbial safety enhancement of microgreens under LED and different lighting systems

Microgreens share similar traits with full-sized fresh produce and sprouts, which has shaped the place these crops have in terms of legislation. Currently, there are no safety guidelines that are specific to microgreens in the United States (U.S.). According to the United States Department of Food and Drug Administration (USFDA)’s Produce Safety Rule (2015), regulatory standards classify microgreens in the same category as mature leafy greens which are generally consumed raw (72). In the absence of microgreen specific safety guidelines, existing frameworks designed for sprouted seeds and leafy greens could be used as a reference by the growers although there are distinctions among these crops (13). The recommended food safety guidelines for microgreen producers under the Produce Safety Rule, recommend the application of required guidelines for sprouts producers. These guidelines include microbial testing for foodborne pathogens such as *L. monocytogenes*, due to the elevated risk that these commodities present (73). However, these guidelines are not mandatory due to the need for further research to aid in understanding the specific food safety risks associated with microgreens. With this consideration, it is essential to establish industry guidelines based on research studies to assist microgreens producers in understanding and adhering to the Produce Safety Rule and controlling the risks associated with microgreens (12).

While the calibration of LED parameters offers a powerful mechanism for biomass optimization and nutritional biofortification, ensuring the microbiological safety of indoor-grown crops remains a critical prerequisite for commercial viability. The warm, humid microclimates and high-moisture growth substrates inherent to CEA systems, provide ideal conditions not only for microgreen development but also for the persistence and proliferation of foodborne pathogens. Addressing these safety challenges requires a multi-faceted understanding of how microbiological hazards enter the production chain and how targeted interventions can mitigate risks without compromising crop quality.

### 5.1 Contamination of microgreens

There are various routes through which foodborne pathogens can enter microgreen production systems, including but not limited to contaminated seeds, water quality, growing medium, improper handling, and storage issues. Throughout the production cycle, microbial contamination poses a persistent risk, as it can be introduced at multiple points via water, soilless substrates, air, or through contact with workers in the case of CEA grown microgreens (72). The majority of research on the food safety risks of microgreens have examined pre-harvest contamination risks. Most studies are done in broccoli, sunflower, and radish cultivars for microbiological assessments, and evaluation is often measured by aerobic mesophilic bacteria counts (AMBC) or by test direct inoculation with pathogens, such as Shiga toxin-producing *Escherichia coli*, *Salmonella enterica* and *L. monocytogenes*, and viral surrogates like Murine *Norovirus* (MNV) and Tulane virus (TV).

Research has shown that most likely pre-harvest contamination risks for microgreens involve transmission of pathogens from contaminated seeds, water, and soilless substrate to the edible part of plants (14, 22, 72, 74–76). The persistence of *S. enterica*, *E. coli* O157:H7, and *L. monocytogenes* on various microgreens cultivars irrigated with contaminated municipal and rainwater note that while high-level contamination (5 log CFU/mL) decreased by 2.5–4.7 log CFU/g on Day 14, lower initial loads (3 log CFU/mL) remained notably more persistent. Furthermore, species-specific survival dynamics were observed, with *L. monocytogenes* typically exhibiting lower persistence compared to the other pathogens tested throughout the sampling period (77). These findings emphasize that microbial quality of irrigation sources is a critical factor for mitigating pre-harvest food safety risks in CEA production systems.

The production system applied during microgreen production has shown to have an impact on the survival of microbial pathogens. A study investigated *E. coli* O157:H7 seed contamination (low −3 to 4 log CFU/g and high −5 to 6 log CFU/g) in radish microgreens compared to bacterial survival rates in peat moss-based soil substitute and hydroponic growth systems (17). During production, significant bacterial survival and growth were observed in both systems, with higher levels in hydroponics. Based on an initial inoculation level of 3.7 (peat moss) and 5.6 (hydroponic) log CFU/g, *E. coli* O157:H7 populations were observed to be up to 5.7 and 5.3 log CFU/g on sample microgreens, respectively. Also, systemic contamination was impacted both edible and inedible parts with seed coats as the major site of pathogen growth (17). While harvested microgreens exhibited significantly lower *E. coli* populations compared to those on sprouts from seeds contaminated at the same initial levels, *E. coli* (O157:H7 and O104:H4) still proliferated during both the sprouting and microgreen phases (16). The growth of *S. enterica* serovars in alfalfa sprouts and Swiss chard microgreens has been observed to be significantly impacted by the inoculation level, the specific growth medium used, and the duration of seed storage (14). Additionally, significant shifts in microbial populations were observed, with microbial changes determined to be lower in microgreens compared to sprouts. Moreover, contamination affected both edible and non-edible parts in Swiss chard microgreens, suggesting that microgreens pose potential systemic food safety risks due to pathogen contamination.

In a study evaluating nine microgreen species, Shiga-Toxin producing *E. coli* (STEC) grew 5 logs over 5 days at 21 °C, colonizing external tissue and in certain instances, colonizing stomata (18). Various factors such as tissue type, contamination route, inoculation dose, humidity, and growth substrate affected the growth of STEC (Sakai) on sprouting seeds. For instance, higher concentrations of STEC (Sakai) were detected on cotyledon with irrigation water when compared to direct seed inoculation (18). Investigating the transmission dynamics of *E. coli* O157:H7, a study demonstrated that pathogen colonization from the spermosphere to aerial plant tissues is highly species-dependent and influenced by inoculation methodology. The study observed that true leaves consistently exhibited lower bacterial colonization densities compared to cotyledons, indicating that plant developmental stage and species-specific host traits are critical determinants of pre-harvest contamination risk (78). Spoilage bacteria, *Pseudomonas* spp., was found to be abundant in microgreens grown in hydroponic systems, and research predicted two-way transmission pathways between the plants and their environment (79). Some studies have shown that the initial pathogen load (low or high CFU) also directly affects the degree of contamination, as well as the subsequent survival and proliferation of these pathogens on microgreens (14, 17, 18). These findings indicate microgreens pose a systemic food safety risk from pre-harvest conditions, that can be closely linked to the growth system.

### 5.2 Mitigation strategies for microgreen safety

Recent studies have evaluated various decontamination strategies during pre-harvest or post-harvest stages to reduce microbial load in microgreens including bacteria (such as pathogenic and non-pathogenic *E. coli*, *Salmonella* spp., and *L. monocytogenes*), yeast and mold, and viruses (75, 80–85). Treatments to reduce pathogens on contaminated microgreens included the use of chemical sanitizers such as chlorine, citric acid, peroxyacetic acid and ascorbic acid, salts such as CaCl_2_ (Calcium Chloride) and MgCl_2_ (Magnesium Chloride), oxidizing agents such as Hydrogen Peroxide (H_2_O_2_), Sodium hypochlorite (NaClO) and ozone water, reducing agents such as Ethanol (E) and application of non-thermal technologies such as UV-C, UV-A, White LED, advanced oxidation process (UV-C/H_2_O_2_) with Micro-Nano Bubbles (MBs) applied individually or in combination at pre-harvest and post-harvest levels (15, 75, 76, 83–92). A recent study indicated that an enhanced Advanced Oxidation Process (AOP) specifically combining H_2_O_2_, UV-C and MBs offers a promising new method for disinfecting seeds in microgreen cultivation (83). While post-harvest UV-C treatment significantly reduced pathogen loads on microgreens, it is an insufficient standalone solution for the inherent food safety challenges due to irregular disinfection on plant tissues and limited reduction (75). Furthermore, alternative light-based strategies, such as continuous UV-A exposure, have shown limited efficacy, failing to provide sustained phytochemical enhancement or microbial reduction beyond the levels provided by the standard white LED light (86). The studies evaluating decontamination techniques on microgreens are shown in Table 2.

**TABLE 2:** Comparison of studies evaluating decontamination techniques on microgreen to various treatment conditions including lighting systems.

Microgreens	Decontamination technique (contamination route and/or treatment)	Microbial target	Treatment conditions	Key findings	Highest inhibition achieved	Reference
Sunflower and radish	UV-C treatment (Soilless substrate contamination)	*Salmonella enterica*, *Escherichia coli* O157:H7 and *Listeria monocytogenes*	Uni and bidirectional UV-C exposure at varying distances and exposure time (Dose: from 0.03 to 2.07 kJ/m^2^), artificial contamination on agriculture perlite (medium)- 10^5^–10^6^ CFU/g	Highest pathogen inhibition with bidirectional UV-C treatment at a 10 cm distance of 120 s. Pathogen regrowth of 0.3–1.7 log occurred during 14 days of storage at 4 °C.	*S. enterica* (3.1 log CFU/g), *E. coli* O157:H7 (3.0 log CFU/g), and *L. monocytogenes* (2.0 log CFU/g)	(75)
Roselle	Advanced oxidation process, AOP (UV-C/H_2_O_2_) combined with Micro-Nano Bubbles, MBs (seed contamination)	Total aerobic bacteria, Total coliform and *Escherichia coli* (*E. coli*)	Treatments include water wash (control), 5% hydrogen peroxide (H_2_O_2_), UV-C (36 watts), advanced oxidation process (AOP; H_2_O_2_ + UV-C), and improved AOP by combination with microbubbles (MBs; H_2_O_2_ + MBs and H_2_O_2_ + UV-C + MBs) on microbial loads on seeds.	Improved AOP treatment (H_2_O_2_ + UV-C + MBs) are more effective than other treatments due to highest hydroxyl radicals.	Yeast and molds (1.0–2.0 log CFU/g) and *E. coli* (2.0 log CFU/g)	(83)
Arugula	Chemical treatment (surface or interior tissue contamination)	Tulane virus (TV) and rotavirus (RV)	Hydroponically grown aragula treated with peracetic acid (PAA) at 30 or 80 ppm for up to 3 min: contamination either externally or internally	Disinfection efficacy: higher on arugula surface for RV, not dependent on location for TV	Rotavirus on surface (5.0 log PFU/g), rotavirus inside (1.5 log PFU/g). Tulane virus on surface and inside (<2.0 log PFU/g)	(89)
Cress, rocket, pea	White light (LED) supplemented with UV-A radiation (natural contamination)	Total aerobic bacteria (TAB)	Microgreens at 1 seed/cm^2^ with growing light/white LED (control) compared to white light supplemented with ultraviolet radiation (treatment)	Total aerobic count and Enterobacteriaceae counts ranging from ∼3.8 to 4.2 log_10_ CFU/g, but significant difference between control and treatment group.	Total aerobic count (approximately 3.74–4.14 log CFU/g)	(86)
Radish	Chemical and ozonated water treatment (natural contamination)	Aerobic mesophilic bacterial yeast and mold count	Treatments include chlorinated water (100 mg L^–1^ NaClO), ozonated water (0.16 mg L^–1^ O_3_), tap water, and an unwashed control.	Ozone water effectively reduced initial aerobic mesophilic bacterial populations with no statistically significant difference to chlorine treatment	Total aerobic count (0.6 log CFU/g)	(92)
Radish	Chemical treatment (pre-harvest soil substrate contamination)	*Salmonella enterica* Typhimurium and *Escherichia coli* O157:H7	Microgreens inoculated with pathogens sprayed with chlorinated water (0.50, 1.00, and 2.00 ppm) 1–4 times during growth.	Pre-harvest spraying could reduce pathogenic load, but is not an effective standalone food safety control measure.	1.1 log CFU/g for *Salmonella*, 0.9 log CFU/g for *E. coli* O157:H7	(76)
Buckwheat	Storage temperature modified atmosphere packaging followed by storage (natural contamination) Post-harvest wash treatment followed by storage	Total aerobic mesophilic bacteria (AMBC)	Storage temperature: 1 °C, 5 °C, 10 °C, 15 °C, and 20 °C. Packaging atmosphere: Polyethylene films with oxygen transmission rates (OTR) of 8.0, 16.6, 21.4, and 29.5 pmol/(m^2^⋅s⋅Pa). Wash treatments: No wash, water wash, 50 mg/L chlorine, and 100 mg/L chlorine for 30 s.	Initial reduction of AMBC but increased growth after 7 days of storage in all wash treated microgreens	AMBC reduced from water wash (0.3 log CFU/g), 50 mg/L chlorine (0.9 log CFU/g), and 100 mg/L (1.3 log CFU/g)	(87)
Chinese cabbage	Chemical treatment (natural contamination and post-harvest wash followed by storage)	Aerobic mesophilic bacteria count (AMBC) and coliforms	Sanitizers [chlorinated water (CI), citric and ascorbic acid mixed solution (CA + AS), Ethanol (E)], packaging films (PE and PP)	CA + E treatment maintained microbial counts equal to or lower than chlorine (CI) throughout storage. PE films could offer greater advantages than PP film.	Total aerobic count (0.2–0.6 log CFU/g)	(88)
Daikon Radish	Chemical treatment (natural contamination and post-harvest wash followed by storage)	Aerobic mesophilic bacteria (AMB) and yeast and mold (Y&M) counts	Temperature during storage, packaging film followed by storage, and chlorine wash treatment	A 100 mg/L chlorine wash reduced microbes by 0.5 log CFU/g but counts rose again after day 7.	Total aerobic count and Yeast and molds (0.5 log CFU/g)	(15)
Broccoli	Chemical treatment (natural contamination and pre-harvest followed by storage)	Total aerobic mesophilic bacteria (AMB)	Sprayed daily with either acidified water (pH 5.6) alone or in combination with one of the following: 1, 10, or 20 mM CaCl_2_ or MgCl_2_, or 5 mM of the calcium chelator EGTA (for 10 days)	The AMBC population declined early in the storage phase, followed by rapid growth for the remainder of the storage period	Total aerobic count (1.5 log CFU/g)	(90)
Broccoli	Chemical treatment (natural contamination and pre-harvest and storage, Post-harvest and storage, Both pre and post-harvest followed by storage)	Total aerobic mesophilic bacteria (AMB), yeasts and molds (Y&M)	Pre-harvest spray includes CaCl_2_ (10 mM), Ca-lactate, or Ca-Amino acid (1–20 mM), post-harvest dip includes Ca lactate (25–100 mM) with chlorine and combined includes pre-harvest CaCl_2_ + postharvest Ca Lactate	Except pre-harvest CaCl_2_ spray (non-dipped) group, all treatments demonstrated an initial decrease in aerobic mesophilic bacterial counts (AMBC), followed by a period of accelerated proliferation for the remainder of the storage duration.	Total aerobic count (1.0 log CFU/g) and yeast and mold (1.2 log CFU/g)	(91)
Vertical hydroponic microgreen (radish, garnet, broccoli, brussels sprouts, cilantro and kale)	Survey (natural contamination)	Multiple spoilage bacteria and potential human, plant, and fish pathogens including aerobic bacterial counts	Hydroponic and aquaponic system, water quality monitoring	*Pseudomonas* spp. in hydroponic microgreens, two-way transmission pathway between plants and their environment.	No inhibition, production system significantly impacted aerobic bacterial counts. Hydroponic microgreens had 7.3–8.6 log CFU/g TAB, while aquaponic microgreens had 3.7–4.0 log CFU/g.	(79)

Advanced oxidation process (AOP) is a method that uses the combination of highly reactive chemical and light treatment to destroy pathogens efficiently. Micro-Nano Bubbles (MBs) are incredibly tiny gas bubbles that are highly efficient at transferring gases (like oxygen or ozone) into water to destroy pathogens. Total aerobic mesophilic bacteria (AMB) is a total count for bacteria that thrives in the presence of oxygen and moderate temperature. Total aerobic bacteria (TAB) is the measure of the total population of oxygen loving bacteria. Paracetic acid (PA) is a chemical sanitizer that is used to reduce microbial load. Oxygen transmission rate (OTR) is the measurement of the amount of oxygen gas that passes through a specific area of a packaging material over a given period. Ethylene glycol-bis(β-aminoethyl ether)-N,N,N’, N’-tetraacetic acid, EGTA is a highly specific chelating agent that binds tightly to metal ions such as Calcium ions (Ca2+).

Mitigation strategies for microgreens have exhibited a wide variety of efficacy in lowering spoilage bacteria or foodborne pathogens. While UV-C irradiation presents by far the best control for foodborne pathogens, with reductions ranging from 2.0 to 3.1 log CFU/g for *S. enterica*, *E. coli* O157:H7 and *L. monocytogenes*, populations increased after treatment during storage, close to initial levels which could be partly attributed to these pathogens performing DNA repair from UV-C damage (75). UV-C has additionally exhibited significant reductions in yeast and molds (1.0–2.0 log CFU/g) and *E. coli* (2.0 log CFU/g) (83). Chemical treatments or sanitizer washes (chlorine or peracetic acid) are commonly applied for microgreens, due to its consistent results, and low cost. In general, these treatments have proven to be more effective on reducing yeast and mold (reductions ranging from 0.5 to 1.2 log CFU/g) and total aerobic bacteria (reductions ranging from 0.2 to 1.5 log CFU/g) (15, 87, 88, 91). Sanitizer washes for pathogens have not achieved reductions as high as UV-C irradiation, with pre-harvest sanitizer sprays exhibiting reductions of 1.1 log CFU/g for *S. enterica* and 0.9 log CFU/g for *E. coli* O157:H7 (76). Sanitizer washes have proven much more effective for controlling viruses, however the efficacy has varied significantly on the location of the contamination. For example, when rotavirus was present on the surface of arugula microgreens prior to sanitation, a 5.0 log PFU/g was achieved. Meanwhile, internalized rotavirus was only reduced by 1.5 log PFU/g (89). Ozone water also presents a viable mitigation strategy, however in microgreens it has mainly been studied for reduction of aerobic bacteria, with low reductions achieved (0.6 log CFU/g) (92).

### 5.3 Antimicrobial treatment and efficacy of microgreen under LED systems

The use of LEDs for antimicrobial treatment centers on photodynamic inactivation, a process where specific light wavelengths predominantly in the blue region (400–500 nm) is absorbed by the endogenous porphyrins in foodborne pathogens that generates reactive oxygen species (ROS), such as singlet oxygen and hydroxyl radicals that cause lethal oxidative damage to microbes (93). For instance, blue light has proven significantly more efficient than green light at reducing wide range of pathogens including *E. coli* O157:H7, *L. monocytogenes*, *Staphylococcus aureus*, *P. aeruginosa*, *Bacillus cereus*, *Lactobacillus plantarum* and *Vibrio parahaemolyticus* (94, 95). Prior research indicates that blue LED irradiation is effective against pathogenic bacteria in both aqueous buffered solutions or food matrices with or without the addition of external photosensitizers, such as chlorophyll and porphyrins, which trigger the inactivation process (96–98). In developed countries, LED systems have a potential to provide a way to improve the safety of fresh produce while decreasing the post-harvest losses of fruits and vegetables (99). Currently, there is a limited number of studies that have investigated the association of LED light (quality, quantity, photoperiod) with the antimicrobial effect in microgreen production and storage system, highlighting a clear knowledge gap that must be addressed with research.

Light Emitting Diode treatments exhibit high reductions similar to UV-C treatments, however there is a lack of studies that exclusively apply LED treatment, without coupling with additional treatments for analyzing impacts on microbial populations. When coupled with UV-A, aerobic bacteria has been reduced up to 3.74–4.14 log CFU/g, indicating its effectiveness when combined with other light treatments (86). A study comparing the effects of LED light spectra (white, blue, red) on kale microgreens observed that the extracts of kale microgreens irradiated with blue LEDs treatment (kale powder sample) for 10 days was most effective against normal pathogens and multi-resistant pathogens (*Bacillus cereus*, *E. coli*, *P. aeruginosa*, *S. aureus*, *Micrococcus luteus*, and *Staphylococcus epidermidis*) in comparison with red and white light (57). This study suggested that while white LEDs favor carotenoid content in kale microgreens, blue LEDs effectively promote higher phenolic concentrations and greater biological activity including antimicrobial efficacy. Another comparable research on red Chinese cabbage microgreens demonstrates that spectral quality in LED significantly affects phytochemical composition and antibacterial activity.

Comparison of blue, red, and white LED treatments revealed that while blue light (450 nm) promotes higher morphological growth and carotenoid accumulation, red light (660 nm) effectively enhances glucosinolate biosynthesis. Furthermore, the increased glucosinolate levels under red light were correlated with heightened antibacterial activity against multidrug-resistant pathogen *P. aeruginosa*, suggesting that spectral modulation can be done to increase certain nutritional components and increase antibacterial efficacy at the same time. This research was based on freeze dried Chinese cabbage microgreen powder sample cultivated under red, blue and white LED illumination for 10 days (100). These highlight the potential for modulated LED light treatment to enhance nutritional and antimicrobial efficacy in microgreens. Likewise, UV and blue LED light could enhance the food safety of hydroponic microgreens by targeting bacteria in the circulating water (13). The current literature offers very few studies evaluating LED-induced antimicrobial efficacy of microgreens, representing a significant knowledge gap for future research which could be conducted in various microgreen types and variations in LED-treatment (quality, quantity, photoperiod).

## 6 Conclusion and future directions

Controlled Environment Agriculture production systems increasingly replace traditional lighting with LED systems to optimize microgreen characteristics, and more research is needed especially with respect to photoperiod in variety of microgreens to strengthen currently obtained results. All these studies suggest that LED lighting, through variations in wavelength, intensity, and photoperiod, significantly affects microgreen growth, phytochemical content, and post-harvest quality. Optimizing light quality and quantity offers a practical strategy to improve productivity, and nutritional quality. Moreover, microgreen nutritional research has primarily focused on the Brassicaceae family, leaving a notable research gap for other plant groups.

The integration of LED technology into microgreen production and storage system can improve characteristics such as growth and yield, nutritional properties, as well as microbiological efficacy with respect to microgreen type. By modulating LED light spectra such as blue and/or red wavelengths, and/or supplementing it with UV LED lights, producers can simultaneously influence microgreen morphology, increase phytonutrients like polyphenols and carotenoids and enhance antimicrobial efficacy in comparison with traditional lighting systems. Most research on LED light focuses on the influence (wavelengths, intensity and photoperiod) on nutritional properties of microgreens and analyzes microgreens from the Brassicaceae family. This research could be expanded to other microgreen groups in the future, to further explore the impacts of these systems. Moreover, there is only few research studies on the influence of photoperiod on microgreen characteristics and nutritional properties, signifying a notable knowledge gap.

Studies have shown that microgreen production faces systemic food safety risks during both pre-harvest and post-harvest stages due to potential pathogen contamination routes. Currently, decontamination techniques in microgreens involve application of chemical sanitizers (such as chlorine), non-thermal treatments (such as UV-C) and oxidative agents (such as ozone water) used individually or in combination. To the best of our knowledge, only one research has applied LED specifically white LED in combination with UV-A for decontamination which has huge potential for expansion in the future studies considering the advantage of LEDs compared to other treatments. The application of LED light, especially blue LED light, could serve as an effective antimicrobial intervention, promoting photodynamic inactivation to reduce pathogenic risks. Currently, very few studies have evaluated antimicrobial efficacy of microgreens with respect to LED lighting and there is no significant study related to the effect of LED lighting to enhance microgreen safety during production and storage. This further highlights knowledge gaps that exist for future research to mitigate food safety risks through LED lighting application. Specifically, little is known about how variations in LED light quality or quantity or photoperiod may influence microbial populations, pathogen survival, or post-harvest safety during microgreen production and storage.
